# Meeting report from the first meetings of the Computational Modeling in Biology Network (COMBINE)

**DOI:** 10.4056/sigs.2034671

**Published:** 2011-11-30

**Authors:** Nicolas Le Novère, Michael Hucka, Nadia Anwar, Gary D Bader, Emek Demir, Stuart Moodie, Anatoly Sorokin

**Affiliations:** 1 EMBL-EBI, Hinxton, CB10 1SD, UK; 2 Division of Engineering and Applied Science, California Institute of Technology, Pasadena, CA , USA; 3 General Bioinformatics Limited. Reading, UK; 4 The Donnelly Centre, University of Toronto, Toronto, Ontario M5S, Canada; 5 MSKCC - Computational Biology Center, New York, NY, USA; 6 Eight Pillars Ltd, 19 Redford Walk, Edinburgh, UK; 7 School of Informatics, University of Edinburgh, Edinburgh, UK; † Contributed equally to the manuscript

## Abstract

The **Computational Modeling in Biology Network (COMBINE**), is an initiative to coordinate the development of the various community standards and formats in computational systems biology and related fields. This report summarizes the activities pursued at the first annual COMBINE meeting held in Edinburgh on October 6-9 2010 and the first HARMONY hackathon, held in New York on April 18-22 2011. The first of those meetings hosted 81 attendees. Discussions covered both official COMBINE standards-(BioPAX, SBGN and SBML), as well as emerging efforts and interoperability between different formats. The second meeting, oriented towards software developers, welcomed 59 participants and witnessed many technical discussions, development of improved standards support in community software systems and conversion between the standards. Both meetings were resounding successes and showed that the field is now mature enough to develop representation formats and related standards in a coordinated manner.

## Introduction

At the beginning of the 21st century, the life sciences have witnessed a tremendous change in the way research is performed, characterized by the acquisition and analysis of large amounts of quantitative data, as well as the integration of these data within computational models used to understand and investigate living systems. In systems biology, structured representations of information, coupled with rich metadata frameworks and the exchange of knowledge, are fundamental enablers of research. The enormous size of biological data sets and the computational models involved favor exchange and collaboration rather than independent redevelopment.

In computational systems biology, three major efforts to standardize data formats for different knowledge domains stand out by their development models. Each is community-based, with community consultations and democratic processes for choosing the individuals who comprise the editorial boards as well as for making technical decisions during the development of the standards. The Systems Biology Markup Language (SBML) [1], covers computational models of biological processes, describing variables, their relationships and their initial conditions. BioPAX, the Biological PAthway eXchange format [2], enables the representation and exchange of metabolic and signaling networks, and protein-protein and genetic interactions. The Systems Biology Graphical Notation (SBGN) [3] is a set of visual languages enabling the graphical representation of biological processes. All of these formats are being developed by vibrant and diverse communities of developers, theoreticians and end-users. Critical to their interoperability is the existence of common technologies for encoding metadata and enhancing the semantics of the formal representations, including MIRIAM URIs (unique identifiers used in the guidelines for the Minimum Information Requested in the Annotation of Models) and the Systems Biology Ontology [4]. Other more focused or more recent efforts to develop standard formats include, CellML [5], NeuroML [6], and SED-ML [7].

These various standardization efforts were originally developed in independent and largely uncoordinated ways. This has resulted, in some unfortunate redundancy in topical coverage, human effort, and funding. In response to this, many of the individuals involved in the different efforts created the **Computational Modeling in Biology Network (COMBINE [**8**], )**, an initiative whose goal is to coordinate the development of the various community standards and formats in computational systems biology and related fields. By doing so, the federated projects can develop a set of interoperable and non-overlapping standards covering all aspects of computational modeling, at every scale, in every field of biology, in a manner that is similar to how the World Wide Web Consortium (W3C) develops standards for the Web.

One of the first goals of COMBINE has been to coordinate the organization of common meetings. Two different annual meetings are currently being organized. The COMBINE annual meeting replaces the SBML and SBGN fora. COMBINE is an open event targeting not only developers, but end users, it offers the opportunity to hear and make presentations about implementations of standard support, scientific investigations made possible by their use, and exploration of new approaches. The second annual meeting, the Hackathon on Resources for Modeling in Biology (HARMONY), is a hackathon-type gathering primarily targeted at software developers, with a focus on continued evolution of the standards, their interoperability, and supporting software infrastructure.

## First COMBINE annual meeting, Edinburgh, October 6–9 2010

The first annual meeting of the COMBINE organization was held in the Informatics Forum at the University of Edinburgh (UK), as a satellite of the 11^th^ International Conference on Systems Biology (ICSB). The agenda, presentation materials, audio recordings, video recordings of presentations are available on the COMBINE 2010 website. A total of 81 people attended the 14 plenary sessions and breakouts, collectively presenting 42 talks and 30 posters. Most of the presentations and posters have been made part of a special Nature Precedings collection for COMBINE 2010 [9] Day 1, Physiome standards (Peter Hunter, chair)

Peter Hunter, from the Auckland Bioengineering Institute (New-Zealand), briefly described the current state of CellML [10] and FieldML [11], two structured representation formats used in the physiology modeling domain [12]. He then presented the new Physiome Model Repository [13], which features the ability to accommodate a large diversity of model types. Poul Nielsen, from the same institution, elaborated on the modularity features of CellML 1.1, and how to build new models  using a model library [14]. David Nickerson, also from the Auckland Bioengineering Institute, showed how the storage of modular models using the physiome model repository (PMR2) can help with the development of multiscale models, using the example of the nephron. Alan Garny, from the University of Oxford (UK), shared his plans for the future of OpenCell [15], a modeling and simulation application able to use CellML files [16].

### Day1, Simulation Experiment Description Markup Language (Frank Bergmann, chair)

Frank T. Bergmann, from the University of Washington (USA), presented the Simulation Experiment Description Markup Language (SED-ML [17], ) [7] and the tools he developed to support SED-ML including the .net based API library libSedML [18,19]. Richard Adams, from the University of Edinburgh (UK), presented the Java API library JlibSEDML [20] and its support in SBSI [21], the modeling infrastructure being developed by the Centre for Systems Biology at Edinburgh [22]. Ion Moraru, from the University of Connecticut (USA), presented further developments of jlibSEDML that enhance the functionality of the library.

### Days 1 and 2, Visual representations (Stuart Moodie, Alice Villéger and Ralph Gauges, co-chairs)

Three sessions were dedicated to the visual representation of models. The first was on the current status of the languages forming the Systems Biology Graphical Notation [3,23]; the second focused on software support for SBGN, and the third session concerned non-SBGN efforts to represent graphs.

Nicolas Le Novère, from the EMBL-EBI (UK), reported on the status of SBGN Entity Relationships and proposed extensions, in particular for the representation of nested entities (domains) [24]. Stuart Moodie, from the University of Edinburgh, presented the status of SBGN Process Description and its evolution and changes that have been proposed for inclusion in Level 1 Version 2 of the specification. These include changing the semantics of complex subunits, changing the state glyph, and adding an annotation glyph [25]. Huaiyu Mi, from the University of Southern California (USA) provided an update on SBGN Activity Flows and a discussion on the problem of units of information carrying the molecular nature of the activity bearer [26].

Bernard de Bono, ( EMBL-EBI), closed the session by discussing the representation of anatomy and an attempt to use SBGN Entity Relationships to represent physiological mechanisms. The SBGN issues outlined during this session were the topic of dedicated breakout sessions during the evenings of days 2 and 3. These breakouts were deemed very productive.

The SBGN session closed with the announcement of the winners of the first annual SBGN competition. The software system SBGN-ED [27] was awarded  the prize in the category “*Best SBGN software support - Completeness, exactitude, validation*”. The category “*Best SBGN software support - Layout and rendering*” resulted in a tie, between Arcadia [28] and VISIBIOweb [29]. The prize for “*Best SBGN map: Breadth, accuracy, aesthetics*” was awarded to the central metabolism diagram of Falk Schreiber and his collaborators from the Leibniz Institute of Plant Genetics and Crop Plant Research (IPK), Gatersleben (Germany). The “*Best SBGN outreach: lecture, training, publication, book, website*” prize was awarded to a tutorial given by Anatoly Sorokin from the University of Edinburgh.

The SBGN software support session was opened by Alice Villéger, from the University of Manchester (UK), who presented the status of LibSBGN, a Java API for manipulating SBGN  [30,31]. Tobias Czauderna, from IPK Gatersleben presented SBGN-ED, a plug-in for the software package Vanted, (Visualization and Analysis of Networks containing Experimental Data, [32] Dragana Jovanovska, from INRIA (France), described SBGN as  supported by BIOCHAM (the Biochemical Abstract Machine) [33,34].

Discussions of graphical visualization continued on day 2 of COMBINE 2010. Ralph Gauges, from the University of Heidelberg (Germany), presented the SBML Level 3 package to encode graph layout and rendering [35]. Huaiyu Mi presented his group's efforts to support BioPAX in CellDesigner [36] and to use CellDesigner notation to convert SBML into BioPAX [37]. Andrei Zinovyev, from the Institut Curie (France) presented a presentation on the behalf of Fedor Kolpakov, from the Institute of Systems Biology of Novosibirsk (Russia), describing the encoding of graphical representation in BioUML [38].

### Day 2, Interactions and reactions (Henning Hermjakob, chair)

A common source of discussions, misunderstandings and disagreements in the COMBINE community is the relationships between interactions and reactions (or processes). This session covered related efforts in the field and was aimed at providing material for more informed discussions. Henning Hermjakob, from the EMBL-EBI presented the efforts of the Human Proteome Organization's (HUPO) Proteomics Standards Initiative (PSI [39]) towards the standardization of interaction data and the iMEX collaboration for their curation [40]. Martin Golebiewski, from the Heidelberg Institute for Theoretical Studies (HITS, Germany), presented SabioML, the format developed to exchange chemical kinetics experiment results [41]. Emek Demir presented the problem of integrating pathways derived from many different data resources, and how Pathway Commons [42,43] aims to tackle the problem with the help of BioPAX [2,44]. David Croft, from the EMBL-EBI, ended the session with a presentation of the new developments in the Reactome pathway database [45], such as its use of SBGN.

### Day 2, Semantics (Camille Laibe and Neil Swainston, co-chairs)

The afternoon of the second day was devoted to the topic of semantics in computational models. It began with a session on the resources available to annotate models. Nick Juty, from the EMBL-EBI, presented an update on the Systems Biology Ontology [46], describing the new links between mathematical expressions and quantitative parameters [47]. Camille Laibe, from the same group, described the planned evolution of MIRIAM Resources [48], including the identification of versions of a dataset, the development of a non-curated branch, and the resolution of annotations in datasets presented in different formats [49]. The session was closed by Andrea Splendiani, from Rothamsted Research (UK), who described mechanisms for encoding further semantics in BioPAX [50]. He also described the activity of the BioPAX workgroup Semantic Web/Linking/Controlled Vocabularies.

A second session of the day was dedicated to the use of the resources presented above. Neil Swainston, from the University of Manchester, described the SBML Level 3 package dedicated to annotation [51]. This package allows, the annotation of SBML attributes (and not just elements, which is all that SBML’s basic  scheme supports), the annotation of annotations, and the expression of a wider variety of annotation relationship types, including negation and alternatives. Ron Henkel, from the University of Rostock (Germany), presented a novel method for model database search and retrieval; the approach ranks the results based on weights assigned to different types of model annotations, the models’ ontological similarities, and user-specified importance of individual query terms given at the time of search [52]. Wolfram Liebermeister, from Humboldt University in Berlin (Germany), then presented the latest developments in SemanticSBML [53], which allows users to annotate models, cluster them, and retrieve and rank models based on their annotations, either using a model, or another type of annotated dataset such as gene expression [54,55].

### Day 3, Conversion and multi-standard software support (Martijn van Iersel and Lucian Smith, co-chairs)

The morning of the third day centered on conversion among standard representations and software supporting several COMBINE standards.

Martijn van Iersel, from Maastricht University (the Netherlands), presented the PathVision 9 pathway vizualization tool  [56,57] and the complementarity WikiPathways community pathway curation system  [58,59], both of which now  support BioPAX format. Jean-Baptiste Pettit, (EMBL-EBI) demonstrated the Systems Biology Format Converter [60], a modular Java framework supporting the development and maintenance of converters between formats such as the COMBINE standards. Alice Villéger shared her experiences in creating a system capable of automatically generating SBGN maps from SBML models and extending those models with the resulting layout.

Lucian Smith, from the University of Washington, discussed the problems of modularity and model composition in relation to CellML and SBML encoding [61]. Camille Laibe described the new generation of model repository [62] proposed as the backbone of BioModels Database, the repository of published models [63,64]. Henning Hermjakob presented the PSI Common Query Interface [65], a software system  supporting queries of multiple resources for protein-protein interaction data expressed in PSI-MI [66]. Augustin Luna, from the National Cancer Institute (USA), discussed software for  handling Molecule Interaction Maps [67], a form of Entity Relationship diagram for representing biomolecular processes. Andrei Zinovyev discussed BiNoM, a Cytoscape plugin for constructing, querying and analyzing biological networks [68,69]; BiNoM imports and exports BioPAX and SBML, and displays the networks in SBGN. On the behalf of Fedor Kolpakov, Andrei closed the session with a presentation on BioUML, a modeling platform that supports nearly all the community standards discussed at COMBINE 2010.

### Day 3, BioPAX (Emek Demir, chair)

The afternoon of the third day started with a session devoted to BioPAX. Emek Demir, from the Memorial Sloan-Kettering Cancer Center (MSKCC, USA), presented new features in  BioPAX Level 3 and compared it to the former widely-used BioPAX Level 2. Nadia Anwar, also from the MSKCC, detailed the new community and governance structure of BioPAX. Gary Bader, from the University of Toronto (Canada), presented his views for the future of the standard. Martijn van Iersel summarized ongoing discussions in the layout workgroup. Emek Demir closed the session with a discussion on generic physical entities and interactions, extending the debate to SBML and SBGN, which face the same problems.

### Day 3, Gaps in coverage and concepts (Robert Cannon, chair)

The third day closed with a session centered on the problems that are not yet covered by the current standardization efforts involved in COMBINE. Nicolas Le Novère presented his vision of what the COMBINE mission is, working towards a comprehensive set of descriptive standards for modeling biology [70]. Robert Cannon, from Textsensor Ltd (UK), discussed his proposal for NeuroML [71]; one of its principle features is explicit typing of model elements [72]. Alexander Mazein, from the University of Edinburgh, proposed a few extension to SBGN Process Descriptions, to better cover enzymatic reactions used in metabolic network representations [73]. Robert Muetzelfeldt, from Simulistics Ltd (UK), described a generic approach for representing complex structures in biological models, based on UML descriptions [74]. Tom Freeman, from the University of Edinburgh, closed the session with a presentation on an alternative to SBGN, the modified Edinburgh Pathway Notation (mEPN), and a demonstration of BioLayout Express^3D^, a 3-dimensional graph viewer.

### Day 4, SBML (Mike Hucka and Sarah Keating, co-chairs)

The last part of the COMBINE meeting focused on SBML [75]. The first session centered on the development of SBML Level 3. The second session was dedicated to SBML software support.

Michael Hucka, from the California Institute of Technology (USA), presented the current status of SBML Level 3 [76] and the ongoing efforts to develop packages to extend the capabilities of Level 3. Jim Schaff from the University of Connecticut Health Center (USA) introduced the SBML Level 3 Spatial Package, which covers the description of compartment geometries and the diffusion of entities. Brett Olivier, from the Free University of Amsterdam (the Netherlands) presented the SBML Flux Balance Analysis Package (which has since then been renamed the Flux Balance Constrains package) [77], which aims to add support to SBML for exchanging flux balance and steady-state analysis models. The session was closed by, Lucian Smith, who presented the SBML Hierarchical Composition Package [78] designed to allow SBML models to be composed from submodels and enable the creation of model libraries.

Sarah Keating, from the SBML Team working at EMBL-EBI, presented the current status of libSBML [79,80], and Nicolas Rodriguez, also from EMBL-EBI, then presented an update on JSBML, a native Java SBML API library [81,82]. They were followed by presentations on two end-user tools supporting SBML: iBioSim [83-85], by Chris Myers of the University of Utah (USA) and SBMLsqueezer [86,87], a plug-in for CellDesigner, by Andreas Dräger of the Bioinformatics Center of Tübingen (Germany).

## 10^th^ SBML Anniversary Symposium

The COMBINE meeting proper was followed on the last day by a symposium and celebration to mark the 10^th^ anniversary of SBML. The first draft of the SBML specification was released in August 2000 by what would later become the SBML Team. Ten years later, a whole ecosystem of tools, teams and research projects has blossomed around SBML, and significant participants of this adventure were invited to give presentations on this occasion.

The symposium was opened by Hiroaki Kitano, from the Systems Biology Institute of Tokyo (Japan), who, a decade earlier and with funding from the Japan Science and Technology Corporation (JST), initiated the project from which SBML eventually emerged. At this anniversary event, Kitano presented his thoughts on the conditions that made possible the emergence of SBML as a successful worldwide standard. He then described his Garuda project to expand the community software development approach to the entire spectrum of computational modeling activities. After Kitano’s presentation, Pedro Mendes, from the University of Manchester, gave an overview of the earliest attempts to develop quantitative models in biochemistry, encode them, and simulate them using computers. Mendes  was one of the earliest contributors to SBML. and Prior to SBML, he contributed to the design of a portable file format for metabolic network models (known as PMB). Hamid Bolouri, from the Fred Hutchinson Cancer Research Center (USA), was the head of the initial SBML Team at the California Institute of Technology beginning in 1999. His presentation focused on CRdata, a software platform for computational systems biology using R and the Amazon cloud [88]. Herbert Sauro, from the University of Washington, was one of the first members of the SBML team along with Michael Hucka and Andrew Finney, and also worked on PMB. Sauro presented a standardization effort for synthetic biology (SBOL, [89], and its implementation in software tools from his own group, in particular TinkerCell [9].

The symposium resumed after a short break with John Doyle, from the California Institute of Technology. Doyle was the Principal Investigator on a subcontract of the JST grant of Kitano awarded to Caltech and hosted the SBML team from late 1999 into the early 2000s. He presented a summary of his ongoing work in applying control theory to physiological modeling, and expressed the need for better theory and tools to connect physiological measurements to an understanding of the functioning of the human body. After Doyle's presentation, Andrew Finney, now at Ansys (UK), another early member of the SBML team. described some of the ways in which SBML development worked, or sometimes did not work, and what could learn for the future development of standards. Michael Hucka, the third member of the original SBML Team and program lead and global co-ordinator since 2004 recapitulated the history of SBML development, some of its successes, and reminded the audience of many important contributors to the evolution and success of SBML. Finally, Nicolas Le Novère, who was one of the earliest contributors to SBML and a developer and contributor since 2004, closed the symposium. . He placed SBML within a broader constellation of emerging standards in systems biology, and called for continued and expanded collaboration within COMBINE.

## First HARMONY, New York City, April 18–22 2011

The first HARMONY meeting was hosted by the Computational Biology group of the Memorial Sloan-Kettering Cancer Center [90] and held at the Rockefeller Research Laboratories building in Manhattan. A total of 59 people attended plenary sessions and technical breakouts over five days. The first day was dedicated to tutorials on the standards and software library implementations facilitating use of the standards. During each of the next three days, a plenary session was devoted to one of the main COMBINE standards (SBGN, BioPAX and SBML). Throughout the day, people held general discussions on the main topic of the day or, in small parallel breakout groups, on the other COMBINE standards. Separate small rooms were dedicated to breakout sessions and *quiet hacking*, because experience from previous meetings has shown that hackathon attendees can neither contain their vocal enthusiasm nor their desire to continue pushing on technical problems regardless of the schedule. The fifth and final day of HARMONY was shared between presentations on SED-ML [7] and issues common to all COMBINE standards; the latter ranging from the use of controlled vocabularies to issues of community organization.

### Day 1, tutorials and poster session

The meeting opened with an introduction to the day from Michael Hucka, followed by overview presentations about SBML, BioPAX and SBGN given by principal developers involved in the development of each standard. The rest of the day was structured into short introductory tutorials on five topics, with materials made available on the meeting website. The first tutorial was on PaxTools; Emek Demir gave an overview presentation of the Java API library supporting BioPAX. This was followed by Nadia Anwar who gave an introduction to the RDF and OWL technologies used by BioPAX, along with a hands-on session using the W3C SPARQL query language to access data from BioPAX files. Tutorials continued in the afternoon,, beginning with a LibSBGN session given by Martijn van Iersel and Tobias Czauderna and served as an update of the current progress and future plans for LibSBGN. It also provided an opportunity to present the initial milestone release of SBGN-ML. this was followed by a libSBML tutorial by Sarah M. Keating and Frank T. Bergmann. Like other sessions in the day, it included illustrative hands-on exercises for attendees to try on their laptops. The day was closed with a tutorial by Nicolas Rodriguez on JSBML.

Following the tutorial sessions, the HARMONY meeting officially opened with a simultaneous buffet reception and evening poster session. In all, 15 posters were presented. They covered a wide variety of topics—everything from databases that can export data in the various formats discussed at HARMONY, to new software tools and emerging standards, including BioPAX and CellML.

### Day 2, plenary session on SBGN

The first plenary session focused on SBGN. It was chaired by Falk Schreiber and focused on unresolved issues in the 3 SBGN sublanguages: Process Description (SBGN-PD), Entity Relationship (SBGN-ER) and Activity Flow (SBGN-AF). The session started with an overview and history of the SBGN standard from Nicolas Le Novère. This was followed by a report on the status of SBGN-PD by Stuart Moodie and discussion of items that were scheduled to be included in the Level 1 Version 2.0 release of SBGN-PD, but which still remained contentious. These problematic topics included the rules of subunit naming, the exact semantics of the “Empty Set” glyph, resolution of the community vote about reversible arcs, and the use of the SBGN Unit of Information to describe the cardinality of multimers and the material type of EPNs. A record of these SBGN-PD issues is available online at [91].

The next session of the morning concerned SBGN-ER and was led by Nicolas Le Novère. Discussions focused on several issues, particularly the semantics of an “entity” and whether different outcome glyphs were required to describe occurrent (e.g. an interaction itself) and continuant (e.g. a complex resulting from the interaction). Huayu Mi then presented the status of SBGN-AF, which was nearing a maintenance release (Level 1 Version 1.1). The major topic of discussion was whether a separate phenotype and perturbing agent activity were required, and whether SBGN-AF needed different glyphs. In addition, the outcome of the vote on how the type of activity (macromolecule, complex etc.) was indicated in AF [92].

### Day 3, plenary session on BioPAX

The plenary session of the third day was devoted to BioPAX. Gary Bader began with an overview of the planned activities, then introduced the projects and issues that were of interest within the community. The goal was nucleation of interested parties to begin discussions and work. This was followed by the session on specification and data. It was chaired by Emek Demir, who gave an overview of data integration and normalization and discussed progress since his last presentation on the topic during COMBINE 2010. Arman Aksoy then introduced the Patch algorithm and outlined the goal to test data integration at the meeting. He reported that several data providers tested integration with specific data sets. Peter D’Eustachio followed with an introduction to current issues in data exchange, including provenance, use of pathwayOrder and nextStep classes, and multiple organism pathways. He also sought to gather end users of BioPAX to highlight known issues and collect additional issue reports from the community. The ultimate goal was coordinated creation of a list for proposal and specification changes and best practices to be co-ordinated into a working group. This followed with a discussion about future development and ideas for BioPAX level 4.

The specification/data session was followed by presentations about works in progress from various BioPAX working groups which were introduced to the community by the  BioPAX editors. Proposals from the SemWeb working group and the layout co-ordinate exchange group were announced as ready for community feedback. Several working groups met in the afternoon to organize and prioritize their activities.

A session about software tools followed. Software developers discussed current developments in BioPAX Software Tools, specifically the BioPAX validator, the PaxTools API, the PathwayCommons resource and the Chibe Visualization tool [93]. In this context, the attendees also discussed proposed software improvements and updates, bugs in the BioPAX validator, and rules and best practices used by the validator. The BioPAX plenary session ended with a list of action items and next steps that were delegated to editors, working groups, and the core BioPAX developers. The action items were later discussed and prioritized during breakout sessions in the afternoons of the third and fourth days.

The day closed with lecture by Chris Sander (director of the Computational Biology group at the Memorial Sloan-Kettering Cancer Center in New York City, USA) on the analysis of pathways to characterize cancers, predict outcomes and suggest therapeutic avenues.

### Day 4, plenary session on SBML

The morning of the fourth day was devoted to SBML. Michael Hucka began with a review of SBML Level 3 and an update on the statuses of various Level 3 package development efforts.  Many of the Level 3 packages have been highly anticipated by the SBML community and Hucka announced that software implementations of several were available for libSBML. Sarah Keating presented a status update on libSBML version 5, a modular version of libSBML that supports extensions for SBML Level 3 package implementations. Nicolas Rodriguez gave a status update on his ongoing work with Andreas Dräger on JSBML. Following that, Andreas Dräger described his own work on a new software system, KEGGtranslator that is designed to convert KEGG pathways to SBML [94]. Martin Golebiewski updated the audience on new developments in SABIO-RK, a free web-based database providing information about biochemical reactions, their kinetic equations their parameters, and the experimental conditions under which these parameters were measured.

Marco Antoniotti summarized the area of multicellular modeling, which is actively pursued by many research groups worldwide yet remains an area that is not well supported by SBML at present. He was followed by Michel Dumontier, who presented exciting work on advanced search and reasoning over SBML model annotations using semantic web technologies. The final session of the morning provided updates on the development of two SBML Level 3 packages. James Schaff discussed the spatial geometry package for SBML, which supports the representation of models and processes that have non-homogeneous spatial qualities. Brett Olivier discussed his collaboration with Frank Bergmann on a package to support the representation of flux balance constraint models (sometimes also known as flux balance analysis models) in SBML.

The remainder of the fourth day was devoted to parallel discussions on SBML and BioPAX topics.

### Day 5, interoperability and governance

The final day began with a session about SED-ML. Frank Bergmann discussed the SED-ML specification, some pending issues about SED-ML, and libSEDML, an API library for developing software support for SED-ML. Frank was followed by Richard Adams, who presented several SED-ML developments: JlibSEDML, a Java-based complement to libSEDML; a new SED-ML Editor; and a SED-ML web service which includes a validator. These presentations were then followed by David Nickerson, who described the work of the CellML group with SED-ML, and the status of CellML software simulators and model repositories. This was followed by presentation by Nicolas Le Novère about recent developments in KiSAO, the Kinetic Simulation Algorithm Ontology [95], in particular the creation of an OWL version of KiSAO.

The following session explored efforts to foster greater coordination between the governance and outreach activities of the main standards involved in COMBINE today. Robin Haw opened the session with a presentation about community outreach activities. He was followed by overview presentations by the principal organizers of the SBGN, SBML and BioPAX efforts, covering the current governance structures of those organizations. The audience was then engaged in a general discussion about the possibility of creating common governance guidelines that could be shared among the various COMBINE efforts as well as used as templates for other, future standardization efforts that aimed to follow in the footsteps of BioPAX, SBML and SBGN.

The rest of the day consisted of various meetings of scientific advisory boards, editorial committees, additional hacking sessions and ad hoc get-togethers organized by HARMONY attendees.

## Community feedback

Immediately following both the COMBINE 2010 meeting in October, 2010, and HARMONY in April, 2011, we conducted exit surveys of the participants. We used a commercial electronic survey tool and made the questions anonymous, in order to maximize the chances of getting unfettered and honest feedback. We asked similar questions on both occasions. The number of responses to the exit survey for each meeting was approximately 40%. The feedback from the COMBINE survey was available prior to the HARMONY meeting, and we took the results into consideration when organizing HARMONY.

### What could have been done differently?

The feedback from the surveys showed that attendees found breakout sessions very useful; however, the organization of such sessions needs to be improved in future COMBINE and HARMONY meetings. COMBINE and HARMONY were purposefully structured with a combination of formal talks and focused breakouts, though with fewer breakouts during COMBINE. Nevertheless, the organization, management and communication of breakout schedules was deemed suboptimal in the end—respondents complained not only when interesting breakouts clashed with the main session, but also that participants were not always made clearly aware of which sessions were being held, leaving people unsure about how best to split their time. While we attempted to consider this and improve how breakouts were organized in HARMONY, the fact that the same issues were raised again in the HARMONY exit survey shows that we need to revisit this problem in future meetings.

COMBINE respondents raised the suggestion of using parallel sessions in order to help provide more time to the different major efforts, and we implemented this idea in HARMONY 2011, where each main standard was given a specific day and other standards had optional parallel sessions. There was no specific agenda for the parallel sessions from the meeting organizers; instead, the communities were left to organize themselves. This appeared to work well and made the meeting both informative and productive for participants. However, the feedback in the exit survey suggested that a more explicit agenda, such as that provided at COMBINE, would actually have been useful. In the future, we can improve this aspect of the meetings through better coordination between the people organizing the different main topics.

The HARMONY exit survey also highlighted the need to improve the communication about activities at the meeting. HARMONY was intended to be relatively free-form, with little scheduled time, to allow each standard group to self-organize based on the participants and skills actually present on the days in question. For the most part, the feedback suggests that this worked well for people who could organize themselves, but less well for others—especially newcomers—who were not intimately involved in the different communities. Better communication of the schedule and breakouts by the organizers was proposed in the feedback, and this should help in the future to encourage more cross-community collaboration at these meetings.

### What worked well and should be repeated?

Generally, the feedback suggested that the concept of the two different meetings worked well. Most participants understood the structure and what was expected of them at each of the meetings. Feedback suggested that the initial meetings had a different dynamic from the individual standard meetings, and that this also worked well, as it promoted interaction among the standards groups. Given the freestyle organization of a hackathon, participants commented that this helped them focus and make excellent progress on tasks that would otherwise have taken months to achieve.

The tutorials were deemed a good idea, though with some mixed reviews. Some attendees, particularly those actively involved with the different standards, did not find the tutorials useful, while others found the tutorials extremely informative, enabling less active users of the standards and newcomers to “catch up”. To newcomers, the tutorials also were meant to introduce the main individuals involved in each of the standardization efforts but respondents indicated that this could have been achieved in introductions instead. We conclude that tutorials should be kept, and the best improvement to make for future tutorials is to clarify the target audience as well as provide the tutorial contents in advance so that people can determine for themselves whether attending those sessions would be useful.

### HARMONY-specific feedback, what did participants achieved at the meeting

Attendees reported having made progress in specific areas and the availability of dedicated time for particular topics with specific individuals enables progress and resolution of outstanding issues faster than usual. More generally, participants found the meeting  helped them become more familiar with other standards and could see how they could participate more actively. Respondents commented on their ability to discuss and get feedback on issues so they could be be implemented/resolved immediately, and having focused time to do this saved considerable time overall. Thus, the meeting accelerated progress that would otherwise have taken much longer, through e-mail and conference calls.,. Participants overwhelmingly agreed that the COMBINE and HARMONY meetings are informative and productive.

## Conclusions and perspectives

Combining the meetings of the various standardization efforts in computational systems biology was a gamble and could have been a challenge. Indeed, while the communities largely overlap at the level of tool development, the end-users are often different, as are their expectations. Contrary to our worries, the meetings were resounding successes with an attendance and an enthusiasm exceeding our most optimistic hopes. The outcomes were numerous. COMBINE 2010 allowed members of the different communities to become acquainted with the  efforts of others. The extent of the overlap and potential for synergy was very clear for all attendees. In addition to a better coordination of the development of standards, this led to discussions and collaborations that further unfolded at HARMONY 2011. These meetings have been defining moments and hopefully launched an irreversible process. The benefits of coordination and synergy are such that stopping the movement would be a significant blow to the various participating projects. However, there is a downside. The scale of the meetings is different (two to three times larger than the separate fora used to be). At that level, the organizational and financial aspects become very significant and require greater commitment on the part of organizers and attendees alike. Nevertheless, combining these meetings also brings an economy of scale that we believe should be recognized by funding bodies who used to support the efforts separately. The next round of meetings. COMBINE 2011 took  place in Heidelberg from September 3 to 7 2011, after the 12^th^ ICSB [96] at the Heidelberg Institute for Theoretical Studies (HITS). The second annual COMBINE meeting did cement was is becoming the common infrastructure for standardization in systems biology. The next HARMONY hackathon will take place in Maastricht from May 21 to May 25 2012. The third annual COMBINE meeting will be a satellite of the 13th ICSB in August 2012 in Toronto.
